# A Liquid Hydrogel to Restore Long Term Corneal Integrity After Perforating and Non-Perforating Trauma in Feline Eyes

**DOI:** 10.3389/fbioe.2021.773294

**Published:** 2021-12-15

**Authors:** Alejandro Juarez, Mohamed Djallali, Marilyse Piché, Mathieu Thériault, Marc Groleau, Sharifa Beroual, Christopher D. McTiernan, Grace Lin, Pierre Hélie, Michel Carrier, May Griffith, Isabelle Brunette

**Affiliations:** ^1^ Department of Ophthalmology, Université de Montréal, Montreal, QC, Canada; ^2^ Centre Universitaire d’Ophtalmologie de l’Université de Montréal à l'Hôpital Maisonneuve-Rosemont, Montreal, QC, Canada; ^3^ Maisonneuve-Rosemont Hospital Research Center, Montreal, QC, Canada; ^4^ Department of Microbiology, Infectiology and Immunology, Université de Montréal, Montreal, QC, Canada; ^5^ Division of Cardiac Surgery, University of Ottawa Heart Institute, Ottawa, ON, Canada; ^6^ Department of Pathology and Microbiology, Faculty of Veterinary Medicine, Université de Montréal, Montreal, QC, Canada; ^7^ Department of Clinical Sciences, Faculty of Veterinary Medicine, Université de Montréal, Montreal, QC, Canada; ^8^ Institute of Biomedical Engineering, Université de Montréal, Montreal, QC, Canada

**Keywords:** cornea, corneal ulcer, corneal perforation, liquid sealant, biomaterials, regeneration, wound healing

## Abstract

**Purpose:** To evaluate long-term *in vivo* functionality of corneas regenerated using a cell-free, liquid hydrogel filler (LiQD Cornea) after deep corneal trauma in the feline model.

**Methods:** Two healthy cats underwent 4 mm diameter stepwise 250/450 µm deep surgical corneal ablation with and without needle perforation. The filler comprising 10% (w/w) collagen-like peptide conjugated to polyethylene glycol (CLP-PEG) and 1% fibrinogen and crosslinked with 2% (w/w) 4-(4,6-dimethoxy-1,3,5-triazin-2-yl)-4-methylmorpholinium chloride (DMTMM), was applied to the wound bed previously coated with thrombin (250 U/ml). *In situ* gelation occurred within 5 min, and a temporary tarsorrhaphy was performed. Eyes were examined weekly for 1 month, then monthly over 12 months. Outcome parameters included slit-lamp, Scheimpflug tomography, optical coherence tomography, confocal and specular microscopy, and immunohistochemistry studies.

**Results:** The gelled filler was seamlessly incorporated, supporting smooth corneal re-epithelialization. Progressive in-growth of keratocytes and nerves into the filler corresponding to the mild haze observed faded with time. The regenerated neo-cornea remained stably integrated throughout the 12 months, without swelling, inflammation, infection, neovascularization, or rejection. The surrounding host stroma and endothelium remained normal at all times. Tomography confirmed restoration of a smooth surface curvature.

**Conclusion:** Biointegration of this hydrogel filler allowed stable restoration of corneal shape and transparency in the feline model, with less inflammation and no neovascularization compared to previous reports in the minipig and rabbit models. It offers a promising alternative to cyanoacrylate glue and corneal transplantation for ulcerated and traumatized corneas in human patients.

## Introduction

The human cornea is the transparent front of the eye that refracts light into the eye for vision. Any disease or damage that causes loss of that transparency will result in vision loss. Corneal ulceration, with or without corneal perforation, often leads to severe complications, including infection, choroidal hemorrhage, retinal detachment, vision loss, and loss of the eye. Non-infectious ulcerative keratitis or perforations were responsible for 16% of the 7,816 stromal or full thickness corneal transplantations performed in the United States in 2019 (EBAA, 2020). Only a limited number of options are currently available to repair ulcerated or perforated corneas (Deshmukh et al., 2020) and even when repaired, these cases carry a poor prognosis due to the elevated risk of rejection, high astigmatism, scarring, and vascularization (Tan et al., 2012), which underlines the need for additional or complementary therapeutic options for these eyes. Cyanoacrylate glue is usually used to seal small perforations. However, it is not devoid of complications and it necessitates the use of a therapeutic contact lens to prevent pain, rubbing, or displacement of the glue. Additionally, cyanoacrylate glue is not transparent and can block vision if within the visual axis. It can also trigger inflammation, scarring and vascularization, and it does not prevent infection. Hence, it is a temporary solution requiring more than one application while waiting for a human amniotic membrane patch or a lamellar or full-thickness corneal graft (Vote and Elder, 2000; Anchouche et al., 2020).

Newly developed polyethylene glycol-based (PEG) sealants show promising results for sealing ocular wounds, but do not address regeneration (Hoshi et al., 2015; Shirzaei Sani et al., 2019). McTiernan et al. (2020) reported the development of a LiQD Cornea consisting of a cell-free liquid hydrogel matrix made of short collagen-like peptides conjugated with polyethylene glycol and mixed with fibrinogen to promote sealing of corneal perforations in animal models. *In situ* gelation occurred spontaneously at body temperature within 5 min. The LiQD Cornea promoted progressive epithelial and stromal regeneration in the rabbit and Göttingen mini-pig corneal models, and also nerve regeneration in the latter (McTiernan et al., 2020). Full-thickness perforations, however, were only evaluated over 1 month in rabbits, a species capable of endothelial regeneration.

The purpose of this study was to verify the capacity of the LiQD Cornea for inducing corneal regeneration, in terms of biointegration and long-term functionality, to repair a deep corneal ablation with or without perforation, in the adult feline cornea, which like human corneas, does not regenerate its endothelium. Understanding the functional regeneration that can be achieved, particularly in adult eyes of a third species, provides the necessary safety and efficacy data needed for extrapolation of results to human subjects ahead of clinical evaluation.

## Materials and Methods

### Preparation of the LiQD Cornea

Synthesis of collagen-like peptide conjugated to polyethylene glycol (CLP-PEG), reconstitution of 10% (w/w) CLP-PEG mixed with 1% fibrinogen and crosslinked with 2% (w/w) 4-(4,6-dimethoxy-1,3,5-triazin-2-yl)-4-methylmorpholinium chloride (DMTMM), reconstitution of thrombin, mixing and application of the LiQD Cornea were achieved as reported previously (McTiernan et al., 2020).

### Surgical Protocol

**FIGURE 1:**
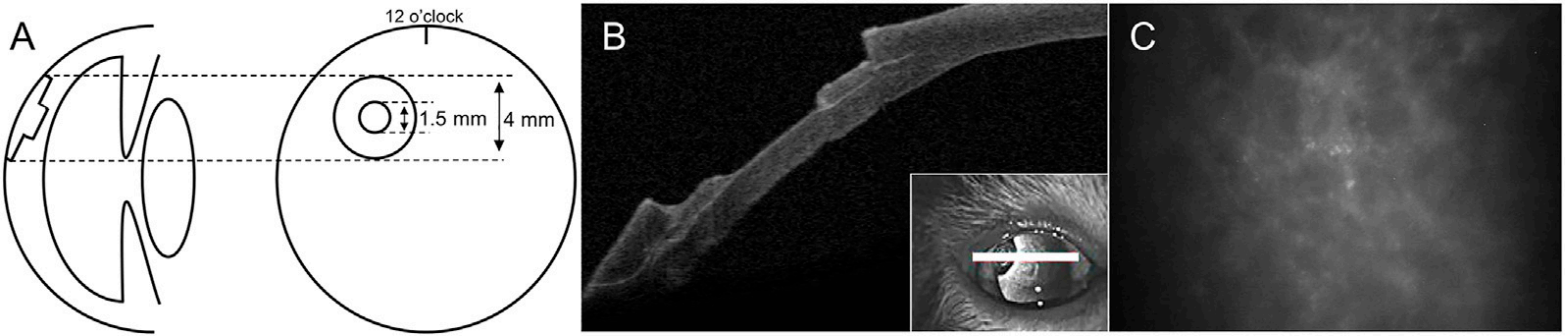
Surgical model. Schematic illustration **(A)** and optical coherence tomography imaging **(B)** of the stepwise ablation profile. Confocal microscopy **(C)** of an acellular gelled LiQD cornea matrix sample 30 min after its delivery from the syringe.

With institutional ethics approval and in accordance to the guidelines of the Association for Research in Vision Science and Ophthalmology (ARVO), corneal surgeries were performed without complications. Two healthy domestic felines aged 12 months and weighting 4.5 and 5.2 kg were obtained from a certified supplier. One eye per animal was randomly assigned for surgery while its contralateral eye served as a control. Surgeries were performed under general anesthesia by the same corneal surgeon (IB). In both animals, a stepwise surgical corneal ablation was performed, consisting of a first 4 mm diameter x 250 µm deep ablation, deepened centrally by an additional 1.5 mm diameter x 200 µm deep ablation (total depth 450 µm) (Figures 1A,B), using dermatology punches (Miltex, Integra, York, PA), a guarded micrometric diamond knife (Meyco, Anton Meyer & Co., Biel, Switzerland), and a Crescent blade (Mani, Tochigi, Japan). In one animal (#A), to simulate perforations observed in the clinic, a 30 gauge needle was used to perforate the wound bed and enlarge the perforation site until it caused a wound leak. These peripheral interpalpebral ablations, with and without perforation, were meant to mimic one of the wound profiles commonly seen in the clinic. The open wounds were then dried with a surgical sponge and pre-coated with thrombin (250 U/ml). They were filled with the LiQD Cornea up to below the surface, as it was felt that an excess of sealant might stimulate foreign body sensation and irritation, which could result in eye rubbing, implant dislodgement and extrusion from the animal eye. *In situ* formation of a gel occurred within 5 min. A temporary tarsorrhaphy of the third lid (nictitating membrane) was then performed to ensure protection of the ocular surface. Figure 1C illustrates the confocal microscopy aspect of the acellular gelled LiQD cornea matrix 30 min after its delivery from the syringe.

### Medication

Animals were preanesthetized with an intramuscular injection of 0.02 mg/kg buprenorphine (Vetergesic, Ceva, ON, Canada), 2 mg/kg ketamine (Narketan, Vetoquinol, QC, Canada) and 0.2 mg/kg medetomidine hydrochloride (Cepetor, Modern Veterinary Therapeutics, Miami, FL). General anesthesia was induced by inhalation of 5% isoflurane and maintained with 1.5% isoflurane, and intravenous rocuronium bromide (Sandoz, Boucherville, QC, Canada) 0.3 mg/kg was used to relieve tension on the globe. At the end of surgery, subconjunctival injections of dexamethasone (1.2 mg in 0.3 ml), cefazolin (55 mg in 0.25 ml), and tobramycin (10 mg in 0.25 ml) were done, and an Elizabethan collar was installed. Animals received oral prednisone steroid, 5 mg daily for 3 days, before and 3 days after surgery.

The postoperative care regimen included diclofenac 0.1% (Diclofenac Ophtha, Sandoz) and moxifloxacin 0.5% (Moxifloxacin, Sandoz) ophthalmic drops twice a day for 4 weeks and dexamethasone/tobramycin (0.3%/0.1%) (Tobradex, Alcon Canada, Dorval, QC, Canada) four times a day until the tarsorrhaphy was removed. Prophylactic oral famciclovir (famciclovir, Sandoz) 125 mg daily was started on admission and continued until the end of the study to prevent feline viral rhinotracheitis. Postoperative examinations were performed under sedation with medetomidine hydrochloride (0.2 mg/kg IM) and atipamezole hydrochloride (Revertor, Modern Veterinary Therapeutics) was used as reversing agent. Animals were euthanized with an intravenous dose of pentobarbital sodium 0.5 ml/kg (Euthanyl, Vetoquinol).

### Clinical Outcome Assessment

Eye examinations were started once the tarsorrhaphy was opened, 14 days after surgery, and repeated weekly for 1 month and monthly for 12 months. They included biomicroscopy (Haag-Streit, Bern, Switzerland) with and without topical fluorescein (Fluorescein sodium ophthalmic strips USP, Hub Pharmaceuticals, Scottsdale, AZ), Scheimpflug Tomography (Pentacam HR, Oculus, Wetzlar, Germany), wide angle optical coherence tomography (OCT) (Visante 1000; Carl Zeiss Meditec, Dublin, CA), Thorlabs OCT (Thorlabs, Newton, NJ), *in vivo* confocal microscopy (Confoscan 3, Nidek Technologies, Vigonza, Italy) (months 4–12), and non-contact specular microscopy (Konan Medical, Nishinomiya, Hyogo, Japan). All of the above instruments were used to assess the biointegration of the implants. The Pentacam HR was used to study the 3D shape of the cornea, and more specifically to monitor corneal surface rehabilitation. It consists of a rotating Scheimpflug camera that generates images in three dimensions. It generates complete corneal imaging in a few seconds and any eye movement detected by a second camera is corrected for in the process, which makes it a very useful instrument to study corneal topography and pachymetry in the animal model. The corneal optical densitometry display also presents automatically generated scatter data, which in reproducible conditions allow to quantified and follow corneal opacification.

### Post-Mortem Assessment

*Ex vivo* confocal microscopy (Rostock Cornea Module/HRT III; Heidelberg Engineering GmbH, Heidelberg, Germany) was performed on enucleated eyes. Corneas were then processed for histology and immunohistochemistry analyses. Keratocyte density and distribution were assessed by measuring the number of Hoechst-stained nuclei per mm^2^ using ImageJ software (http://imagej.nih.gov/ij/provided in the public domain by the National Institutes of Health [NIH], Bethesda, MD, United States). Histology studies were also performed on ipsi- and contralateral retropharyngeal and submandibular lymph nodes.

### Immunohistochemistry

Samples were placed into Optimal Cutting Temperature medium (Tissue-Tek OCT Compound, Sakura, Torrance, CA) and frozen in liquid nitrogen. They were then sectioned, fixed, permeabilized and processed using the antibodies listed in Supplementary Table S1. Representative fluorescent images were acquired from a minimum of 5 sections per protein per cornea (with the exception of CD9 and TSG101, which required only one cryosection per cornea) using a confocal laser-scanning microscope (LSM880, Carl Zeiss Microscopy, Göttingen, Germany) and a widefield fluorescence microscope (Zeiss AxioImager Z2, Carl Zeiss Microscopy, Göttingen, Germany). The three-dimensional reconstructions of extracellular vesicle (EV) and exosome staining using CD9 and Tsg101 respectively were generated in Imaris v9.2.1 (Bitplane Inc., Concord, MA), with intensity thresholds of 75 and 50 for CD9 and Tsg101, respectively, and a minimum voxel threshold of 10. A co-localization channel of CD9 and TSG101 was built using the same intensity thresholds of 75 and 50, respectively. A surface grain threshold of 0.141 μm was used for DAPI stain and 0.1 μm for the colocalization stain.

## Results

### Reepithelization

Opening of the tarsorrhaphy 2 weeks after surgery revealed full re-epithelialization of the filled area by a flat and smooth epithelium in both eyes, except for a 0.5 × 0.5 mm microscopic epithelial defect remaining in animal #B that motivated a repeat of the tarsorrhaphy for an additional 2 weeks in this animal. Apart from a second similar transient recurrence of the epithelial defect occurring 4 months later and lasting 10 days in animal #B, the epithelial surface of both animals remained smooth and healthy throughout the entire study period. The uncomplicated microscopic epithelial defect that occurred twice in the exact same area was linked to the re-injection of the filler shortly after the initial injection at the time of surgery.

### Biointegration and Transparency

The biosynthetic filler remained stably integrated into the host corneas, without signs of wound dehiscence, extrusion, or conjunctival, corneal or intraocular inflammation. The filler did not stimulate corneal neovascularization, corneal edema, or any sign of immune rejection, ocular toxicity or infection.

**FIGURE 2:**
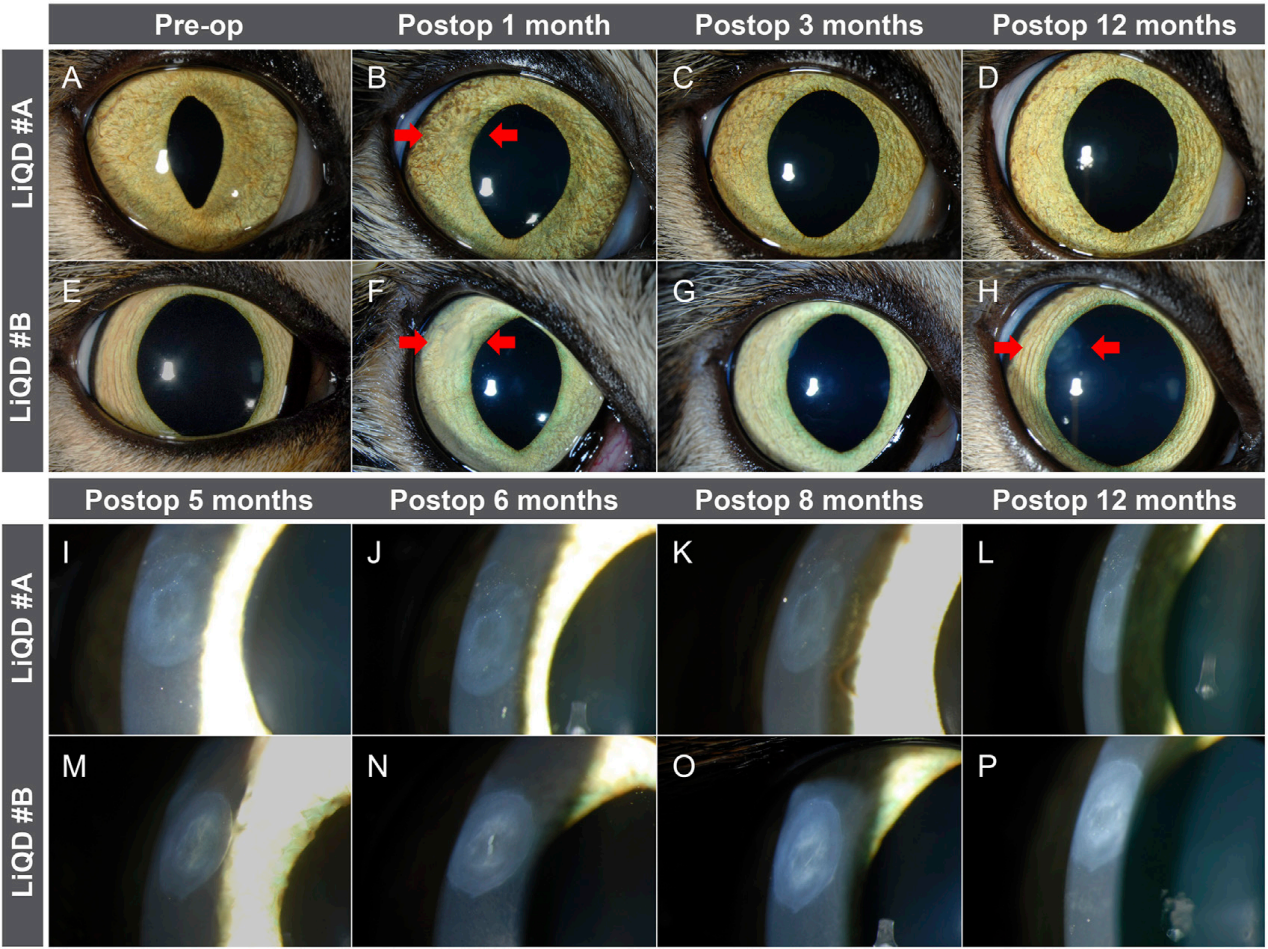
Slit lamp examination. Direct illumination of the implanted corneas before and 1, 3, and 12 months after surgery in animal #A **(A–D)** and animal #B **(E–H)**. Tangential illumination at 5, 6, 8, and 12 months in animal #A **(I–L)** and animal #B **(M–P)** allows to highlight a mild stromal haze at the interface between the filler and the surrounding stroma. Arrows indicate the implanted region.

**FIGURE 3:**
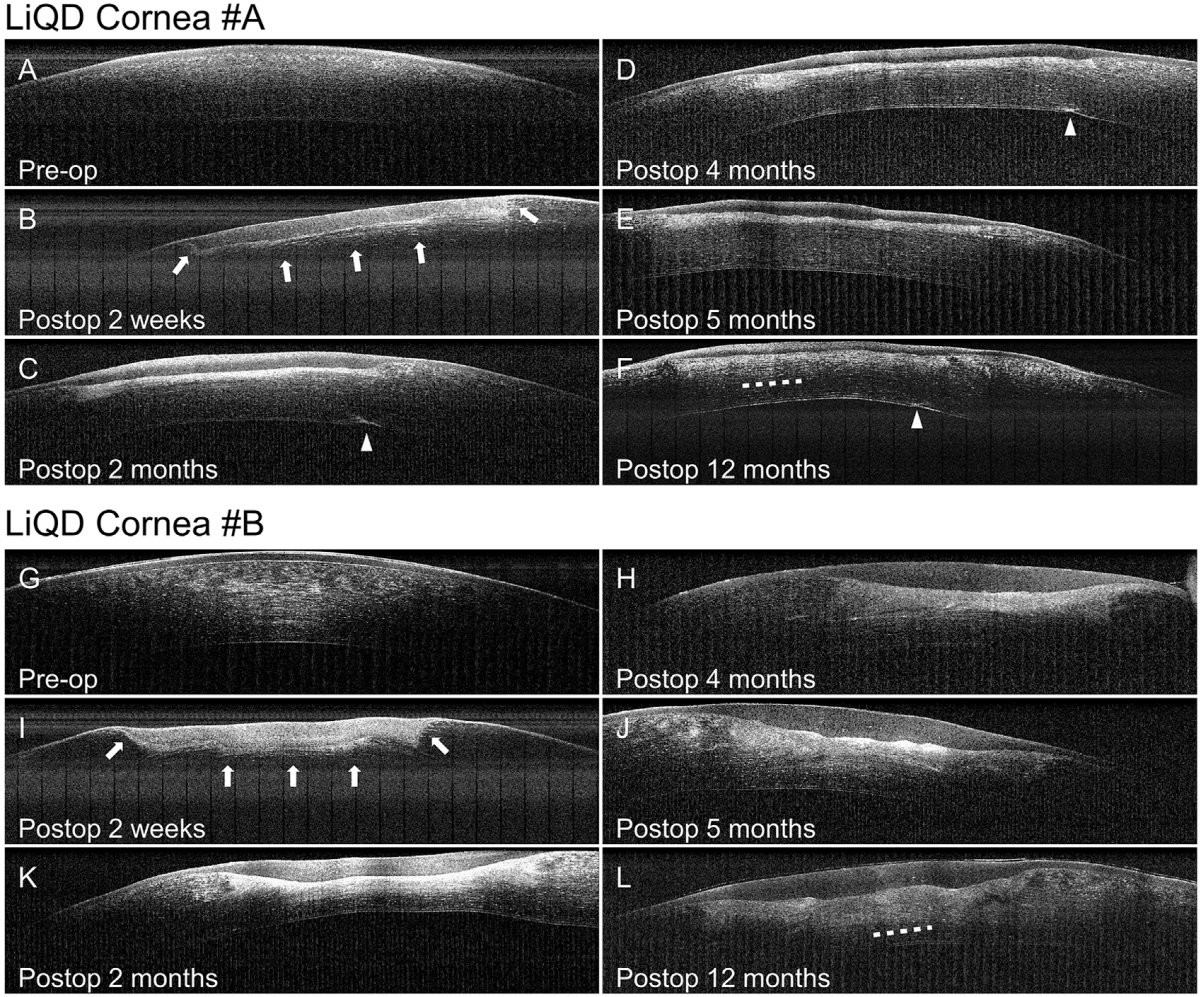
Optical coherence tomography. Progressive replacement of the filler by the regenerated stroma in animal #A **(A–F)** and animal #B **(G–L)**. Arrows indicate the change in density progressing from the interface between the implant and the wound bed toward the anterior aspect of the filler. Arrow heads are pointing to the site of needle perforation in animal #A. Dotted lines indicate the presumed transition zone between the native stroma and the regenerated neostroma.

**FIGURE 4:**
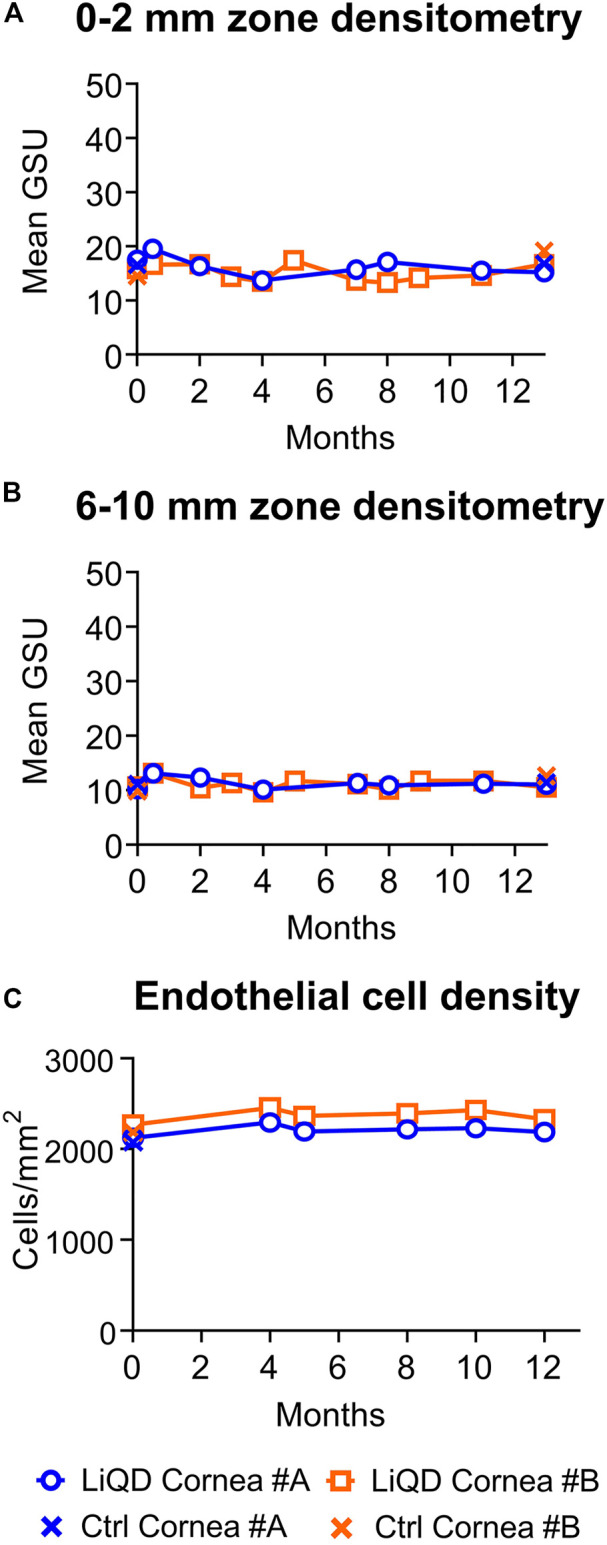
Corneal densitometry. Mean densitometry values in standardized greyscale units (GSU) in the 2 mm diameter central corneal zone **(A)** and in the 6–10 mm diameter peripheral ring shape zone covering the implants **(B)** as a function of time for operated eyes and prior to surgery in non-operated mate controls. Endothelial cell density. Mean endothelial cell counts as a function of time for operated eyes and non-operated mate controls **(C)**.

Transparency of the implanted corneas allowed for visualization of the iris surface fine details at all times (Figures 2A–H). One month after surgery, however, slit lamp examination with tangential illumination revealed a mild stromal haze at the interface between the filler and the surrounding stroma (Figures 2I–P). This transition zone became thicker, smoothing the initial sharpness of the wound edges, as documented by OCT (Figure 3). The stromal haze was studied using the standardized greyscale unit (GSU) of the Pentacam densitometry program, which confirmed in animal #A transient increase in stromal density returning to normal preoperative values at 4 months and remaining stable thereafter. In animal #B, both the clinical haze and densitometry values were denser and faded slower. Overall, the mean densitometry values for the central cornea (radius 0–2 mm) and the 6–12 peripheral ring shape zone covering the implants remained stable, within average values found in human subjects (Ni Dhubhghaill et al., 2014) (Figures 4A,B).

**FIGURE 5:**
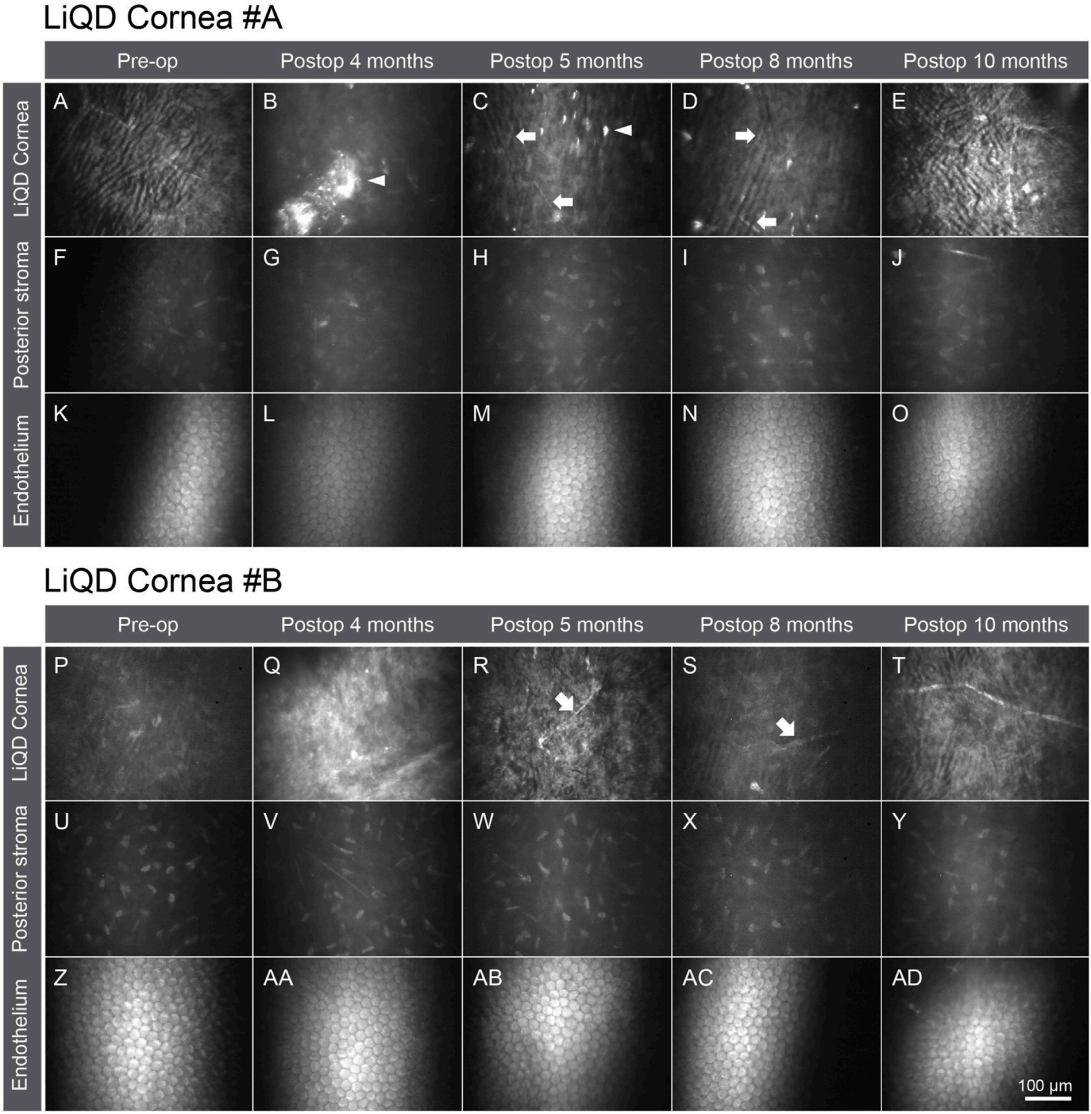
*In vivo* confocal microscopy of animal #A. Normal cornea before surgery **(A, F, K)** and progressive replacement of the LiQD cornea matrix. Emergence of numerous reflective keratocytes within the filler was documented 4 months after surgery **(B)**, as well as fine nerve fibers (arrows) 1 month later **(C)**, along with progressive loss of the highly reflective remnants of the filler (arrow heads) **(B–D)**. At 10 months, the neo-stroma replacing the filler **(E)** looked very much like the preoperative native stroma. The posterior stroma **(F–J)** and the corneal endothelium **(K–O)** remained intact throughout the entire study period. *In vivo* confocal microscopy of animal #B. Normal cornea before surgery **(P, U, Z)**. Filler appearance at 4 months postop **(Q)** showing numerous reflective remnants of the filler. Emergence of keratocytes and nerve fibers (arrows) within the filler at 5 months postop **(R, S)**. At 10 months postop, the implanted stroma returned to a stable state similar to normal **(T)**. The posterior stroma **(U–Y)** and the corneal endothelium **(Z–AD)** remained intact throughout the entire study period.

This mild haze seen within the remodeling tissue corresponded to the in-growth of stromal cells. The haze resolved over time, correlated to the progressive replacement of the cell-free filler by keratocytes and nerves (see below). The host posterior stroma remained unchanged at all times. No significant corneal endothelial cell loss was documented in the implanted eyes (Figure 4C), the <10% difference in cell counts between pre and postoperative values being within the range of normal variability reported for endothelial cell count measurements (Hirst et al., 1989; Lass et al., 2005). Furthermore, there was no additional cell loss in the perforated cornea of animal #A. The mean (±SD) serial cell counts measured in animal #A were: Preop: 2,125 ± 40; at 4 months: 2,294 ± 53; 5 months: 2,194 ± 64; 8 months: 2,218 ± 95; 10 months: 2,232 ± 58; and 12 months: 2,188 ± 73 cells/mm^2^, and in animal #B: Preop: 2,269 ± 13; 4 months: 2,457 ± 27; 5 months: 2,366 ± 21; 8 months: 2,394 ± 12; 10 months: 2,431 ± 19; and 12 months: 2,330 ± 33 cells/mm^2^. Representative serial images of the endothelial mosaics are shown in Figure 5 (K-O; Z-AD).

### Colonization of the Filler by the Host Cells and Nerves

**FIGURE 6:**
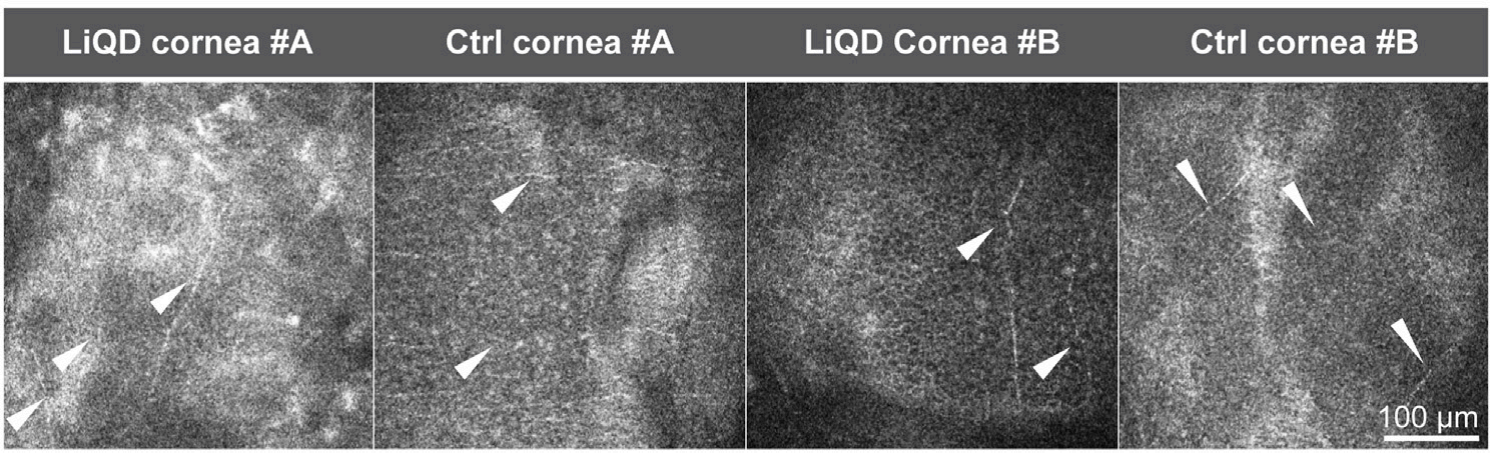
*Ex Vivo* confocal microscopy. Restitution of a sub-basal plexus with numerous parallel bundles of regenerated nerves (arrowheads) similar to those found in the unoperated controls.

*In vivo* confocal microscopy allowed detailed follow-up of tissue remodeling, as illustrated in Figure 5 for animal #A. Emergence of numerous reflective keratocytes within the initially acellular hydrogel matrix was documented at 4 months after surgery (Figure 5B), followed by the progressive emergence of fine nerve bundles 1 month later (Figures 5C–E). Simultaneously, the highly reflective remnants of the filler progressively disappeared (Figures 5B–D). At 10 months post-operation, the regenerated stroma was similar to that of the preoperative native stroma (Figures 5A,E). The integrity of the posterior stroma (Figures 5F–J) and endothelial layer (Figures 5K–O) was preserved at all times. The overall same pattern was observed in animal #B (Figures 5P–AD). Confocal microscopy confirmed the regeneration of a new sub-basal plexus very similar to that seen in the contralateral unoperated eyes in both animals (Figure 6).

### 3D-Corneal Shape

**FIGURE 7:**
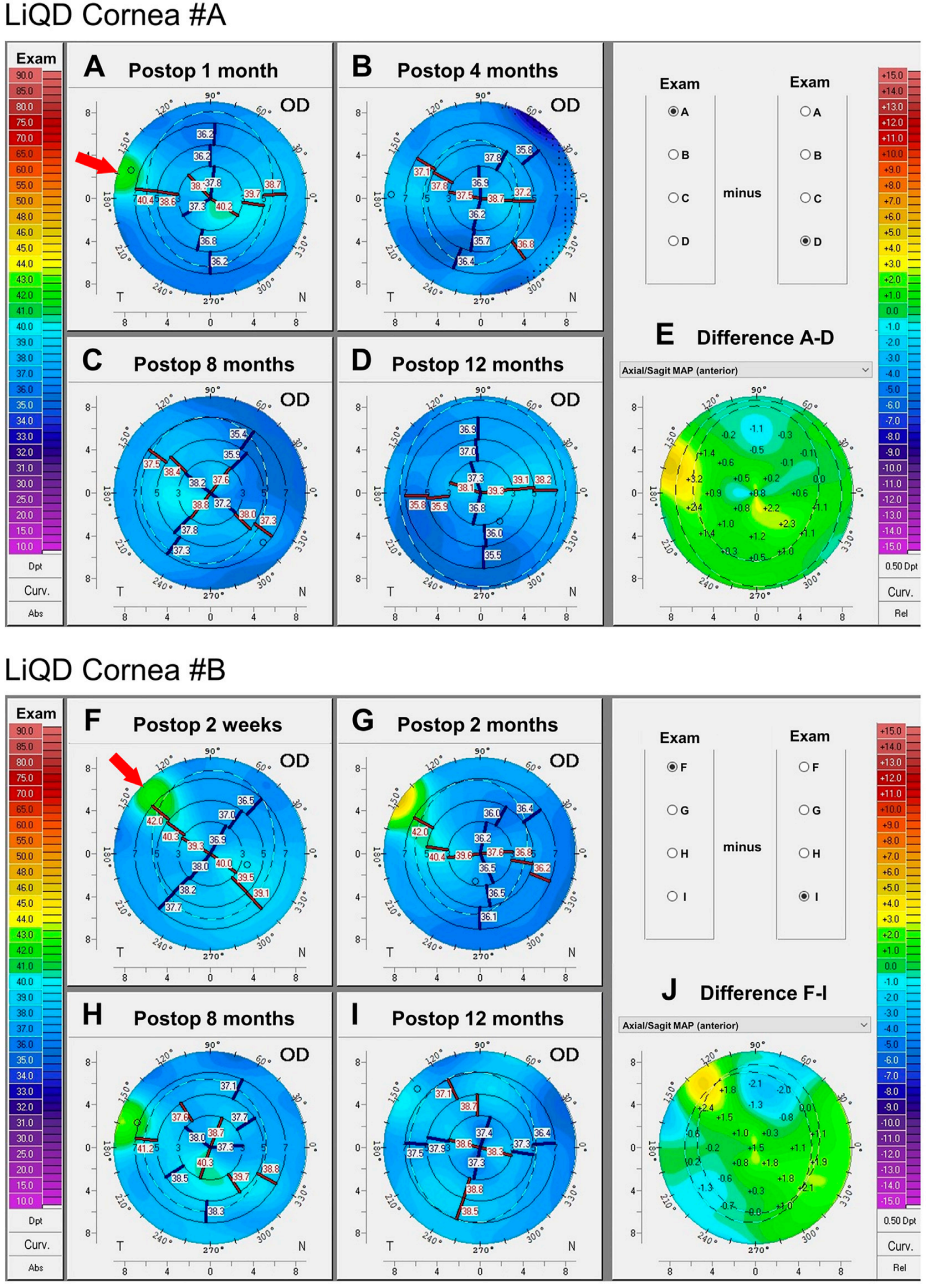
Corneal tomography: Animal #A. Serial sagittal curvature maps of animal #A’s implanted cornea at 1, 4, 8, and 12 months postoperatively **(A–D)**. Smoothing of the corneal anterior surface is summarized by the difference map in **(E)**. Animal #B. Serial sagittal curvature maps of animal #B’s implanted cornea at 2 weeks, and 2, 8, and 12 months after surgery **(F–I)**. Smoothing of the corneal anterior surface is summarized in **(J)**. The arrows indicate the implanted regions.

A mild depression of the peripheral corneal surface was observed at the level of the implanted zone, due to incomplete filling at the time of the surgery, the center of which initially tended to bulge slightly (Figure 7A). This depression was filled by the host epithelium that had grown over, establishing a stable epithelial surface that approximated the normal pre-operative curvature (Figures 7B–D). Restoration of a smooth convex shape most likely resulted from the constant sweeping of the lids over the implanted area during each blink. The same pattern was observed in animal #B, with a slightly slower progression (Figures 7F–I).

The central cornea is the most meaningful in terms of optical quality of the eye, as it covers the visual axis and the center of the pupil. Serial corneal tomographies of the central cornea showed that the main 3D-shape parameters, namely pachymetry, curvature and elevation of the front and back surfaces, were only minimally affected by the filler. Figure 7 illustrates the stability of the front surface central curvature, with astigmatism keratometric values varying only by 0-0.6D for animal #A and 0–0.3 D for animal #B. In the absence of sutures and/or lamellar or full-thickness tectonic graft, filling of these eccentric wounds did not induce traction, edema, scarring or irregular astigmatism susceptible to significantly and/or permanently impact on the optical quality of the central cornea.

### Histopathology and Immunohistochemistry

**FIGURE 8:**
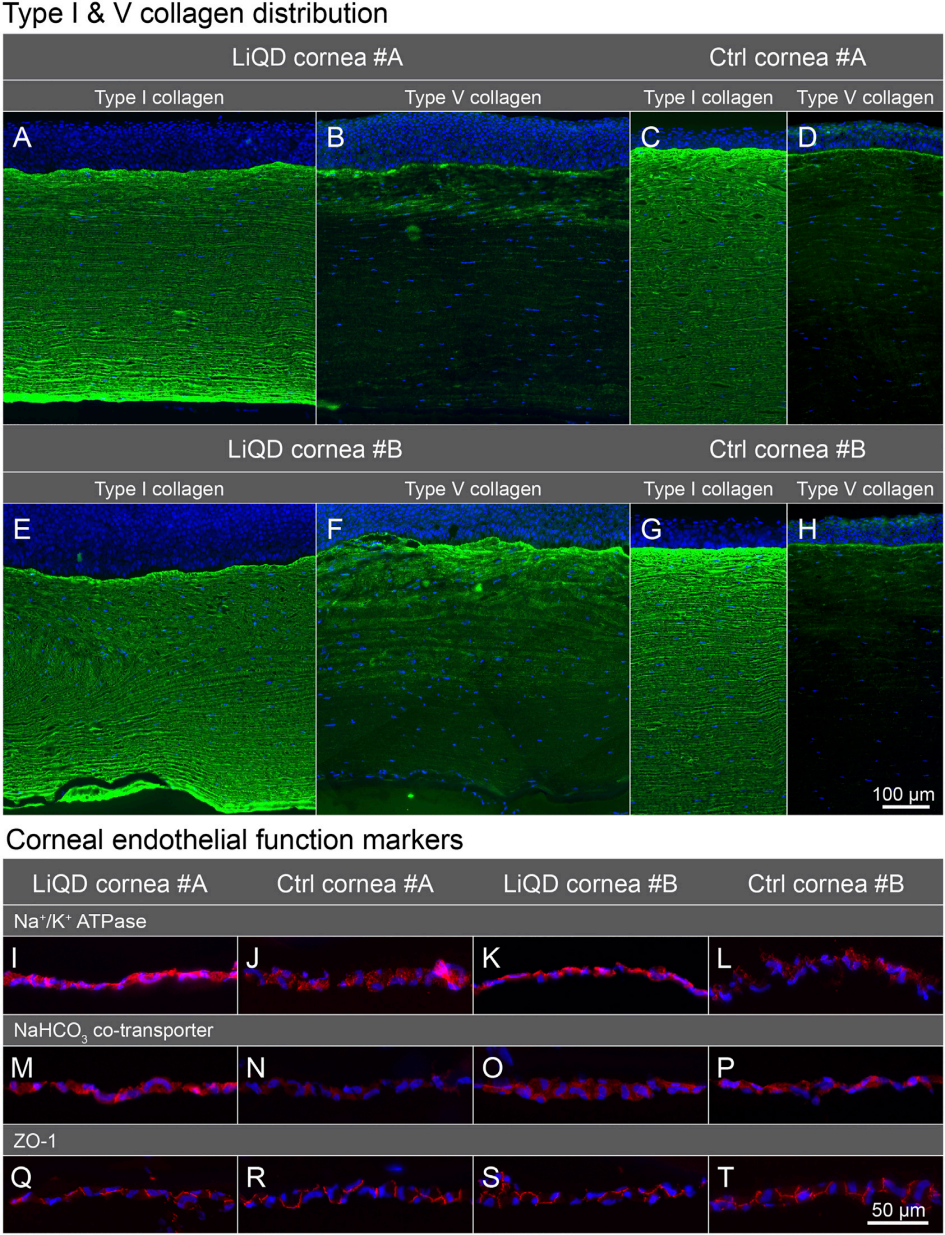
Immunofluorescence labeling. Type I and type V collagen in the LiQD cornea #A **(A–B)**, unoperated control cornea #A **(C–D)**, LiQD cornea #B **(E–F)**, and control cornea #B **(G–H)**. Endothelial cell function-related proteins **(I–T)**: Sodium-potassium pump Na^+^/K^+^-ATPase α1, Na^+^/HCO_3_ cotransporter, and tight junction complex protein ZO-1 were similarly distributed in the endothelium of implanted and control corneas. Cell nuclei were counterstained with Hoechst (blue). Scale bar: 100 µm **(A–H)** and 50 µm **(I–T)**.

Histopathology confirmed the replacement of the filler by a cellular neo-stroma, without inflammatory cell infiltration or neovascularization. The newly formed stroma lamellae were laid parallel to the wound bed. In the anterior part of the neo-stroma, they were less regularly and tightly packed than in the posterior part of the neo-stroma, the architecture of which was closer to that of the unoperated eyes’ native stroma. The expression of type I collagen was uniform and similar to that of control corneas (Figures 8A,C,E,G). The expression of type V collagen was stronger in the neo-stroma than in the native stroma, and especially marked in the sub-epithelial layers, a pattern that was also seen to a lesser degree in the native controls (Figures 8B,D,F,H). Keratocytes were aligned with the collagen lamellae and they did not express the myofibroblast marker αSMA (Supplementary Figures S1A–D). The multilayered epithelium covering the implanted area was thicker than normal but healthy. The corneal endothelium was preserved in all eyes, as confirmed by the unaltered expression of the function related markers sodium-potassium pump Na^+^/K^+^-ATPase, Na^+^/HCO_3_ cotransporter and tight junction complex protein ZO-1 (Figures 8I–T). No remaining PEG was detected in the implanted corneas (Supplementary Figures S1E–H).

**FIGURE 9:**
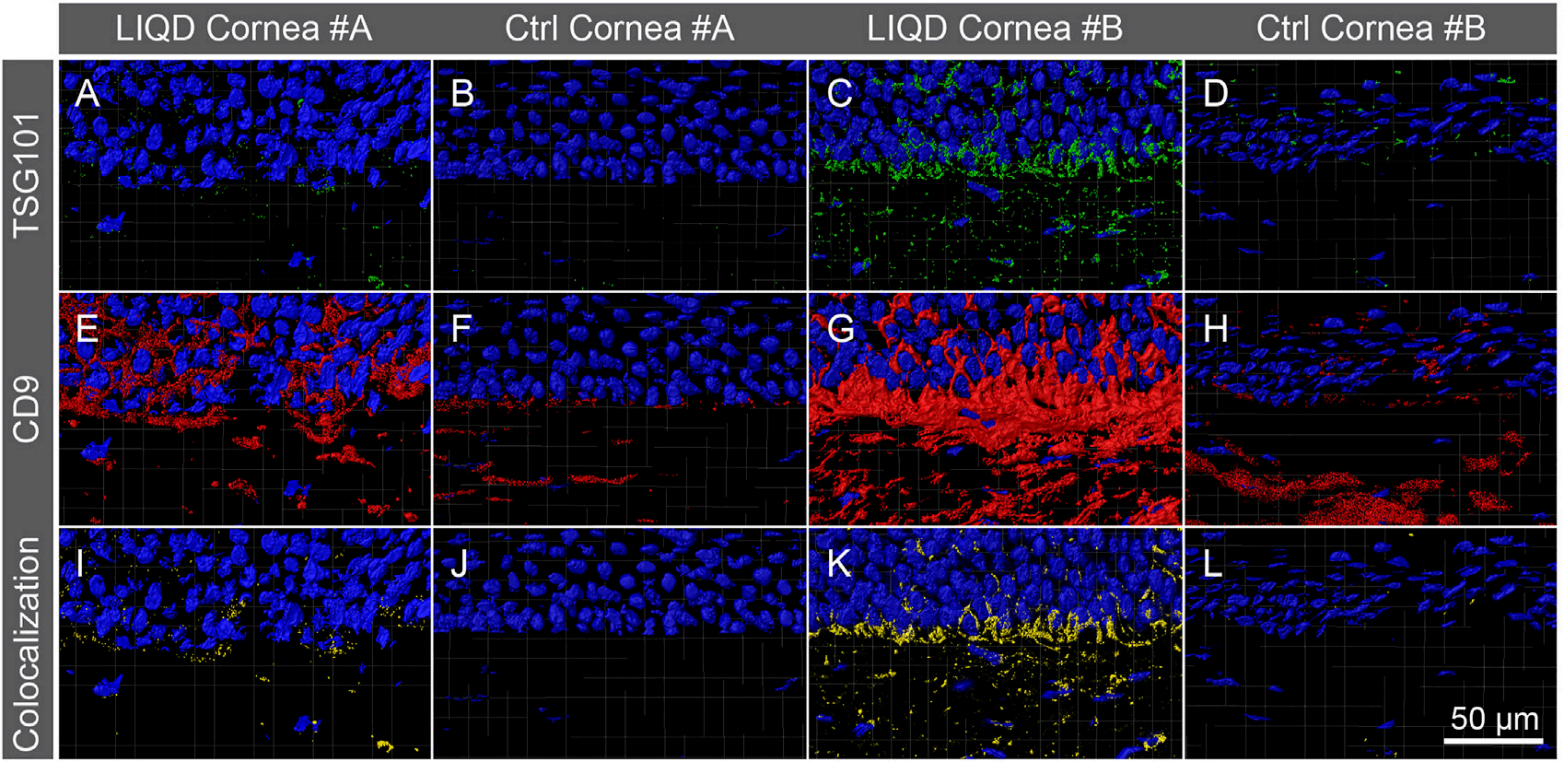
Immunofluorescence of the exosomes. TSG101 **(**green; **A–D)**, CD9 **(**red; **E–H)** and colocalization of both **(**yellow; **I–L)** proteins in the epithelial and subepithelial regions of the LiQD Cornea-implanted and unoperated control corneas of both animals. Surface colocalization reconstruction of TSG101 and CD9 showed a greater abundance of exosomes in the basal layer of the epithelium and sub-epithelial layers of the LiQD Cornea-implanted corneas. Colocalization in the unoperated eyes was minimal. Cell nuclei were counterstained with DAPI (blue). Scale bar: 50 µm.

Immunohistochemical staining of TSG101 showed staining in the epithelium and more diffuse staining in the neo-stroma of LiQD Cornea-implanted samples, whereas the unoperated corneas displayed minimal levels of staining (Figures 9A–D). TSG101 forms part of the endosomal sorting complex required for transport-1 (ESCRT-1) and is a marker for extracellular vesicles (EVs) (Ahmed et al., 2019). The samples were also stained for CD9, another marker for EVs more specific to exosomes (Figures 9E–H) (Théry et al., 2018). Surface reconstructions of the colocalization of TSG101 and CD9 showed that exosomes were present in greater abundance in the basal layer of the epithelium and to a lesser extent in the sub-epithelial layers of the neo-stroma. There was minimal colocalization in the unoperated samples (Figures 9I–L).

The number of keratocytes in the neo-stroma of operated corneas (850 ± 163 nuclei/mm^2^) was increased compared to that of the unoperated contralateral corneas in the corresponding anterior stroma (533 ± 975 nuclei/mm^2^; *p* < 0.001). It was also greater than in the host adjacent anterior stroma (610 ± 113 nuclei/mm^2^; *p* = 0.024) that had cell densities comparable to those of unoperated corneas in the same area (*p* > 0.05). This showed that population of the biomaterial did not deplete the surrounding stroma, nor did it promote keratocytes hyperplasia in the surrounding stroma. No significant differences in cell densities were observed in the posterior stroma of operated and non-operated eyes.

### Lymph Nodes

H&E stained sections from the right and left lateral retropharyngeal lymph nodes of both animals, as well as the right and left mandibular lymph nodes of animal #A showed no activation by the biomaterials (Supplementary Figures S2A–E,G). It was noted, however, that animal #B had a wound in the external ear on the left side. Injury was most likely associated with the observed reactive lymphadenopathy, characterized by follicular hyperplasia (secondary follicles) that was minimal in the right and moderate in the left mandibular lymph nodes (Supplementary Figures S2F, H).

## Discussion

This study confirmed the long-term *in vivo* safety, biocompatibility and functionality of the LiQD Cornea implanted in two eyes of two animals, one with a corneal perforation and the other without perforation. The feline model, the third model on which the LiQD Cornea was tested, presents interesting complementary advantages over the traditional pig and rabbit models previously used, including closer similarities to human corneas in biomechanical properties and thickness (human: 546 ± 41 μm; feline: 583 ± 54 μm; domestic pig: 878 ± 14 μm; minipig: 704 ± 22 μm; rabbit: 387 ± 20 μm) (Dohadwala et al., 1998; Shildkrot et al., 2005; Sanchez et al., 2011; Wang and Wu, 2013; Chen et al., 2015; Telle et al., 2019; Doughty, 2021). Like human corneas, the feline corneal stroma does not swell much, unlike that of the pig. Neither is it as flimsy and prone to collapse as the rabbit cornea (Brunette et al., 2011). Also, feline corneal endothelial cells do not replicate *in vivo*, contrary to species such as the rabbit (Van Horn et al., 1977), making the latter a poor model for assessing endothelial toxicity. Additional advantages over the traditional pig and rabbit models include the fact that the feline head anatomy fits better with human instrumentation, making it easier to estimate the difference with what is routinely seen with human subjects. Interestingly, biointegration of this hydrogel filler allowed stable restoration of corneal shape and transparency in the feline model with less inflammation and without neovascularization compared to previous reports in the minipig and rabbit models. Scarring and neovascularization are the most damaging complications of corneal wound healing.

Corneal transparency and 3D-shape are the two most important clinical outcome parameters to be considered when assessing corneal functionality. The corneal front surface curvature accounts for most of the refractive power of the eye, meaning that any distortion of the front corneal surface (astigmatism) will affect the optical quality of the retinal image and decrease vision. Preservation of a smooth central curvature in the presence of a deep peripheral ulceration treated with the LiQD Cornea, as documented by Scheimpflug tomography, is a promising observation.

While the decision of using a filler such as the LiQD Cornea rather than a corneal graft will depend on the location, depth and severity of tissue loss, it is important to compare the outcome of this procedure with that of traditional techniques, such as patch grafts, penetrating keratoplasty (PK) or deep anterior lamellar keratoplasty (DALK) (Tan et al., 2012; Hos et al., 2019). Peripheral corneal wounds are difficult to treat (Parmar et al., 2009). In a human patient, eccentric wounds similar to those made in the present study would be treated initially with a lamellar patch graft, followed eventually by a larger diameter centered PK or DALK. These surgeries, however, are not devoid of complications, the most frequent being severe, unpredictable and unavoidable surface distortion (irregular astigmatism) caused by the sutures. Other suture-related complications include exposure of loose sutures, infection, ulceration, vascularization along suture tracts, all of which representing known risk factors for rejection (Christo et al., 2001). Most importantly, due to the full thickness severing of corneal nerves, PK and DALK result in corneal anesthesia and poor wound healing, worsening underlying conditions such as ulcerations (Lin et al., 2014). Compromised innervation (or neurotrophic keratitis) is often a causative factor for corneal ulceration. PK and DALK vertical wounds have poor tensile strength, which results in refractive instability, long visual rehabilitation, and even graft dehiscence following minor trauma (Goweida et al., 2015; Meyer and McGhee, 2016). Endothelial cell attrition following PK and graft immune rejection, ultimately causing irreversible graft failure, are also of concern (Ing et al., 1998; Thompson et al., 2003; Cheng et al., 2011; Arundhati et al., 2021). By promoting healthy reinnervation and tissue regeneration while preserving the posterior stromal and endothelial layers, and without stromal dissection, sutures and allogeneic tissue grafting, the LiQD Cornea, could in the future eliminate most of the common culprits encountered with traditional human tissue transplantation techniques. Further surgical indications would have to be weighted against the disadvantages and potential complications overshadowing traditional interventions. The corneal haze seen during *in situ* tissue regeneration in the LIQD Cornea filled wound bed progressively disappeared over time with remodeling. This haze would not be of concern in human subjects for peripheral wounds, even in the early postoperative period. If the wound were more central, encroaching on the visual axis or in the pupillary area, the same haze would temporarily be a source of glare until it fades off, and the haze described herein would not justify corneal transplantation.

Unlike cyanoacrylate glue, the LiQD Cornea is biocompatible and fully biointegrated with time, and in cases of minor and/or eccentric ulcerations, with or without perforation, it could be considered as the final and optimal solution to restore integrity of the globe. At no time the animals showed any sign of irritation from the implant. The consistency of the gelled LiQD Cornea resembles that of the fibrin glue routinely used in human patients to fix conjunctival grafts or human amniotic membranes. Fibrin glue’s surface is typically soft and comfortable, while cyanoacrylate glue is more rigid and irritating for the patient, which can be very annoying and painful especially when it starts to detach. This is one of the reasons why once applied, the cyanoacrylate glue is usually covered with a therapeutic contact lens.

Tarsorrhaphy in this study was used as a painless adjunct therapy to enhance protection of the eye. Tarsorrhaphy is commonly performed in human patients with persistent corneal epithelial defects failing to respond to medical treatments (Dhillon et al., 2020). It can be temporary or permanent and partial or complete, depending on the need. Contrary to animals, LiQD Cornea implantation in human patients would not necessitate a tarsorrhaphy, as simpler alternatives such as increased lubrification or bandage contact lenses would probably be sufficient.

The incomplete filling of the wound at the time of surgery was essentially due to the delivery system used to apply the filler. Future improvement in the delivery system will require greater precision and adaptability to the surgeon’s need. Injection will have to be rapid, as the biomaterial gels quickly. The pressure needed to express the biomaterial out of the syringe and the caliber of the cannula will need to be adjusted according to the filler viscosity while maintaining homogeneity (without air bubbles). In some cases, the use of a mold may also be necessary to optimize shape control with less dependance on epithelial coverage.

It is interesting to notice that stromal regeneration did not occur outside the filler. In the absence of a LiQD cornea scaffold, the ablated stroma did not regenerate into neo-stroma. Where the LiQD Cornea did not completely fill the stroma, the curvature of the cornea was restored by epithelial hyperplasia instead. This suggests that the keratocytes were guided either by the chemistry, the biomechanics and/or the anatomical structure of the filler’s attractive properties, rather than interactions with the epithelium during stromal regeneration. This underlines the importance of paying special attention to the 3D-shape of the injected filler. More work is needed to determine if a second injection of filler might be needed to restore the shape of the cornea.

The extracellular matrix of a healthy corneal stroma is predominantly composed of type I and V fibrillar collagen (Michelacci, 2003; Meek and Knupp, 2015). In this study, the expression of type I collagen in the implanted corneas was uniform and similar to that of control eyes, while the expression of type V collagen was stronger in the recently formed neo-stroma. This is not surprising as type V collagen has an important role in fibrillogenesis. It forms heterodimers with type I collagen to initiates fibril formation and controls fibrils diameter, with its absence being associated with improper fibril assembly and corneal opacity (Wenstrup et al., 2004; Sun et al., 2011). Regeneration of a neo-stroma appears to involve both cells and extracellular matrix.

There was a significant increase in the production of exosomes and EVs near the basal epithelium and the anterior stroma in the implanted corneas, in comparison with the unoperated corneas. This would suggest that exosomes are being secreted from the epithelium to the neo-stroma and could be playing a role in wound healing, as discussed in previous studies (Jangamreddy et al., 2018). This also coincides with the increased expression of type V collagen seen in these implanted corneas, suggesting a joint role in the promotion of a healthy neo-stroma regeneration.

In the healthy adult cornea, keratocytes exhibit relatively low levels of activity, while corneal injury stimulates their activation, proliferation, and migration toward the injured region (Zieske et al., 2001). These cells produce relatively low amounts of extracellular matrix and often initiate transition into myofibroblasts that express αSMA and produce a disorganized extracellular matrix, resulting in scarring and hazing (Mohan et al., 2003). In this study, no αSMA positive cells were seen in the treated area, suggesting that the LiQD Cornea mitigated or did not stimulate the transformation of keratocytes into myofibroblasts. The keratocytes used the LiQD as a scaffolding material onto which they deposited their new extracellular matrix, with minimal impact on corneal transparency.

Finally, no PEG, a major component of the LiQD Cornea, was detected in the cornea after 1 year, confirming the successful replacement of the biomaterial through remodeling.

Some similarities were observed between the stromal regeneration promoted by the LiQD cornea and the tissue-engineered (TE-) stromal grafts previously transplanted by our group in the feline model (Boulze Pankert et al., 2014). The TE-stromal grafts were produced *in vitro* using the self-assembly approach prior to transplantation, while the LiQD cornea neo-stroma was generated *in vivo*, necessitating different implantation techniques. In both cases the implants yielded a transparent cornea allowing visualization of the fine iris detail. However, a discrete haze was visible when viewed under tangential illumination at 4 months post-operation. The 4-months follow-up of the TE-grafts was too short to document the diminishing of this haze as seen in the LiQD corneas. In both cases, the newly formed stroma was slightly less compact than the mature native stroma and it expressed collagen types I and V in similar proportions. In addition, the extracellular matrix secreted and assembled by the activated keratocytes in both constructs yielded neo-stromas that did not clinically resemble scar tissue, as corroborated herein by the absence of expression of αSMA and the absence of inflammatory cells or fibrosis.

This study has its limitations, due mainly to its very small sample size of two animals and the absence of controls other than the preoperative status of the operated eye and that of the healthy unoperated contralateral eye. A sample size of n = 2 is too small to drive any general conclusions, however the purpose of this descriptive study was not to demonstrate safety and efficacy of the LiQD Cornea, as this was already shown in minipig and rabbit models in McTiernan et al. (2020) using unoperated eyes and eyes undergoing anterior lamellar transplantation of syngeneic corneal grafts as controls. The goal of the present study was to demonstrate in a third animal species with corneas surgically and medically closer in several aspects to the human cornea, that the LiQD Cornea sealant can repair deep corneal ablations with or without perforation. Larger controlled studies will be needed to answer several other questions, such as the usefulness of the LiQD Cornea in other types of wounds (e.g., traumatic lacerations, geographic ulcers, perforations larger than 1 mm, infected or not), or the impact of various adjuvant therapies to minimize and eventually prevent haze formation (e.g., serum tears, anti-inflammatory agents, stem cells injections).

The corneal wound sealing and biointegration properties of this hydrogel filler offer a highly innovative and promising alternative to cyanoacrylate glue and other corneal transplantation techniques currently used for ulcerated and traumatized corneas in human subjects. With safety and efficacy demonstrated in three successive models, we are getting closer to clinical transfer. This product could considerably help ophthalmologists and their patients dealing with non-infected corneal ulcerations and/or perforations. Ideally, the LiQD Cornea filler should be applied to ulcerated corneas prior to perforation to limit complications and restore a corneal anatomy as close as possible to normal. Surgical time would be around 10 min, including disinfection and preparation of the patient, followed by a 15-min resting period to ensure that the glue is dry, all of which being significantly less than the 1–2 h intraoperative time needed to perform a lamellar or full thickness corneal transplantation in an operating room, to which the patient has to add all the pre- and postoperative medical care management, as well as administrative process attached to hospitalization. Application of the LiQD Cornea does not require human donor tissue, a cornea specialist, or an expensive medical center with operating rooms, while potentially allowing a better prognosis.

## Data Availability

The raw data supporting the conclusions of this article will be made available by the authors, without undue reservation.
