# Supplementary material for: A Liquid Hydrogel to Restore Long Term Corneal Integrity After Perforating and Non-Perforating Trauma in Feline Eyes
Source: Front Bioeng Biotechnol. 2021 Dec 15;9:773294. doi: 10.3389/fbioe.2021.773294 (PMC8714956; doi:10.3389/fbioe.2021.773294)
Supplement: Supplementary file 1 [file DataSheet1.docx]

Supplementary Material

**Supplementary Table 1 : Antibodies used for immunofluorescence studies.**

| Antibody | Species | Clone | Company (Catalog#) |
| --- | --- | --- | --- |
| Type I collagen^1^ | Mouse | COL-1 | Millipore-Sigma (C2456) |
| Type V collagen^2^ | Rabbit | Polyclonal | Abcam (ab134800) |
| Alpha smooth muscle actin^1^ | Mouse | 1A4 | Abcam (ab5694) |
| Na+/K+ ATPase pump^2^ | Mouse | C646.6 | Millipore-Sigma (05-369) |
| NaHCO3 cotransporter^1^ | Rabbit | Polyclonal | Millipore-Sigma (AB3212-I) |
| ZO-1^1^ | Mouse | ZO1-1A12 | ThermoFisher (33-9100) |
| Polyethylene glycol^3,4^ | Rabbit | PEG-B-47 | Abcam (ab53449) |
| CD9^3,5^ | Mouse | MM2/57 | ThermoFisher (MA1-80307) |
| TSG101^3,5^ | Rabbit | EPR7130(B) | Abcam (ab125011) |
| Substance P^3^ | Rat | NC1 | Millipore-Sigma (MAB356) |
| βIII Tubulin^3^ | Rabbit | Polyclonal | Abcam (ab18207) |
| Rabbit 488 | Goat | Polyclonal | ThermoFisher (A-11008) |
| Rabbit 594 | Goat | Polyclonal | ThermoFisher (A-11001) |
| Mouse 488 | Goat | Polyclonal | ThermoFisher (A-11012) |
| Mouse 594 | Goat | Polyclonal | ThermoFisher (A-11032) |
| Rat 680 | Goat | Polyclonal | ThermoFisher (A-21096) |
| Mouse HRP | Goat | Polyclonal | ThermoFisher (B40961) |
| Tyramide 594 | n/a | n/a | ThermoFisher (B40957) |
| Streptavidin 647 | n/a | n/a | ThermoFisher (S21374) |
| Hoechst reagent 33342 | n/a | n/a | ThermoFisher (62249) |
| DAPI | n/a | n/a | ThermoFisher (D3571) |

1. Sections were fixed and permeabilized using acetone (EMD Serono, Mississauga, ON, Canada) at -20°C for 10 minutes. Antibodies were diluted in 1% BSA. Both primary and secondary antibodies incubated 1-2 hours at room temperature. Hoechst was added to the secondary antibody solution. Slides were mounted in Vectashield Antifade Mounting Medium (Vector Laboratories, Burlingame, CA).
2. Sections were fixed using 4% paraformaldehyde (Electron Microscopy Sciences, Hatfield, PA) at room temperature for 10 minutes, then permeabilized using 0.2% Triton X-100 (Sigma-Aldrich, Saint Louis, MO) at room temperature for another 10 minutes. Antibodies were diluted in 1% BSA. Both primary and secondary antibodies incubated 1-2 hours at room temperature. Hoechst was added to the secondary antibody solution. Slides were mounted in Vectashield Antifade Mounting Medium.
3. Sections were fixed using methanol (Sigma-Aldrich, Saint Louis, MO) at -20°C for 20 minutes, followed by a subsequent permeabilization using 0.3% Triton X-100 for 15 minutes and incubated in 50 mM ammonium chloride in TBS for 30 minutes. Samples were blocked using 5% FBS and 0.01 g/mL saponin for 1 hour. They then were incubated with the primary antibodies overnight at 4°C and with the secondary antibodies for 1 hour at room temperature. Samples incubated in Vector® TrueVIEW™ Autofluorescence Quenching Kit (Vector Laboratories) for 5 minutes then stained with DAPI (5 μg/mL) for 10 min then mounted in Vectashield Antifade Mounting Medium.
4. We used an Avidin/Biotin Blocking Kit (Vector Laboratories, Burlingame, CA). After blocking in 5% FBS and 0.01 g/mL saponin for 1 hour, sections were blocked with avidin for 15 minutes, then with biotin for 15 minutes.
5. We incubated the sections in 3% H_2_O_2_ for 10 min prior to blocking. Following the secondary antibodies, sections incubated in tyramide (ThermoFisher, Waltham, MA) for 10 minutes.

**
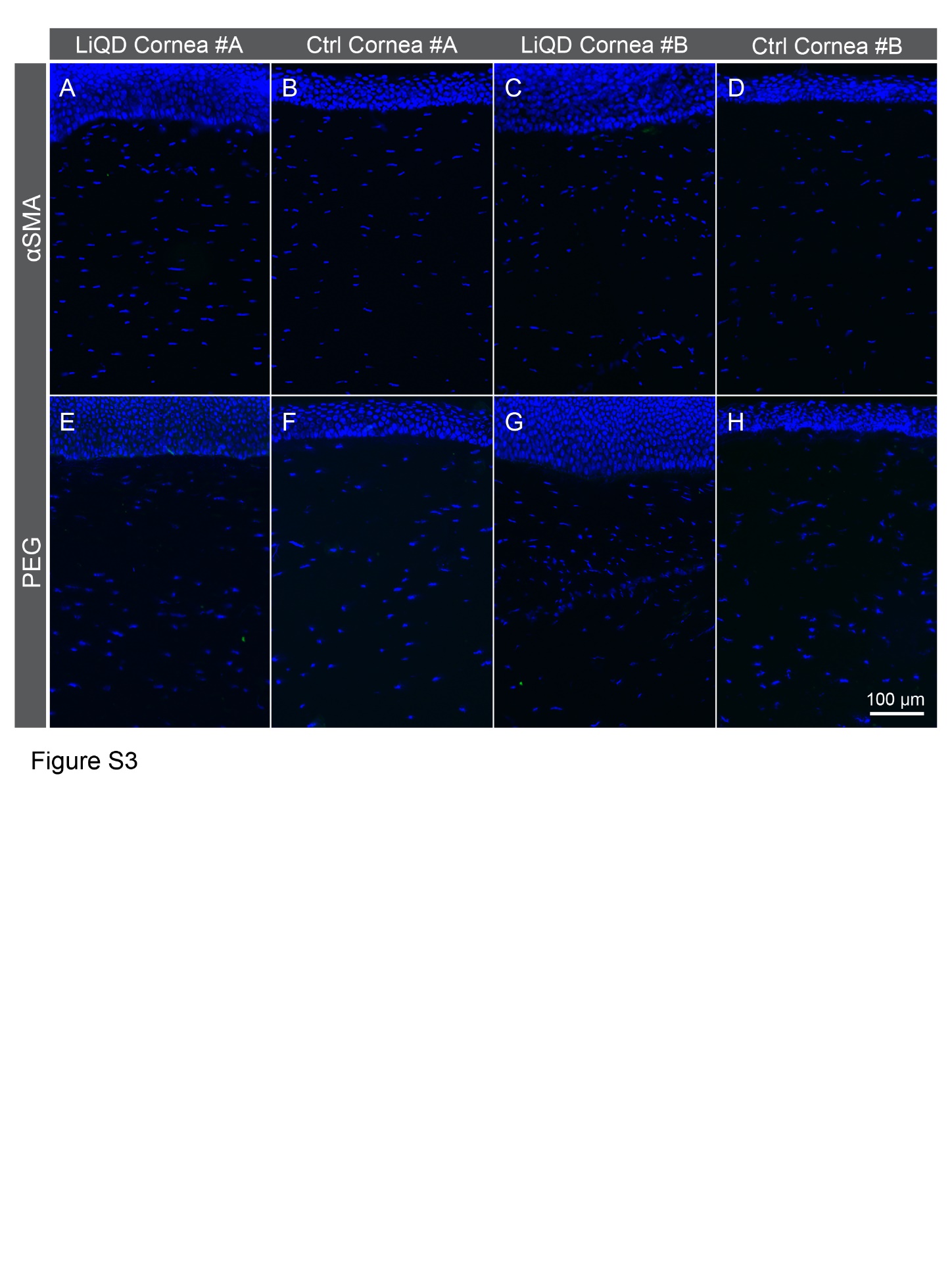
**

**Supplementary Figure 1. Immunofluorescence labeling.** αSMA **(A-D)** and PEG **(E-H)** in the LiQD Corneas #A and #B and their contralateral unoperated control corneas. Cell nuclei were counterstained with Hoechst (blue). Scale bar: 100 µm.


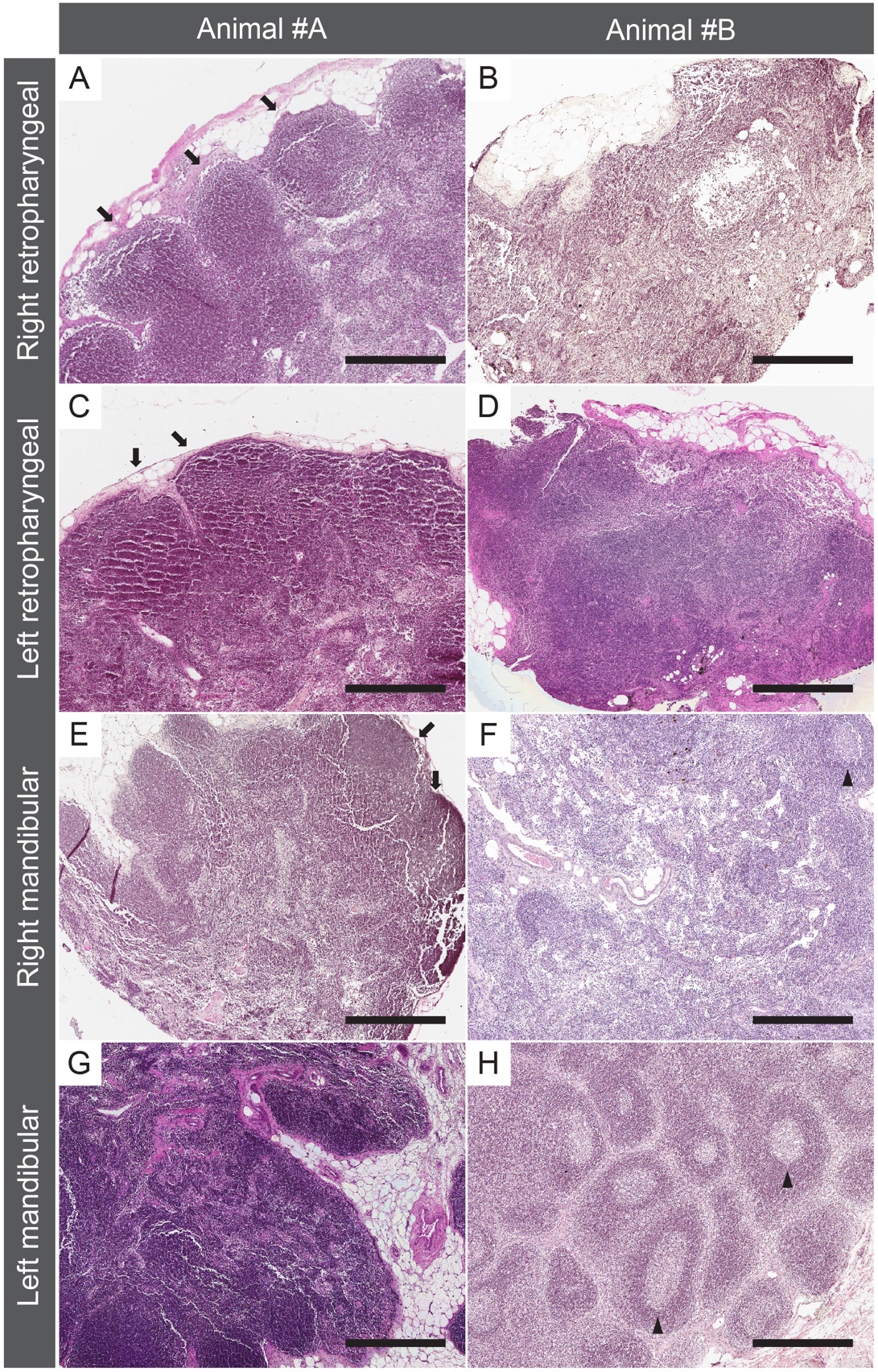


**Supplementary Figure 2. Retropharyngeal and submandibular lymph nodes.** H&E stained sections from the right and left lateral retropharyngeal and mandibular lymph nodes of animal #A (**A, C, E, G**) and #B (**B, D, F, H**). Arrows indicate primary follicles and arrowheads secondary follicles. Scale bars: 500 µm
